# Optimizing feasibility and reproducibility of pacing-induced heart failure model in pigs

**DOI:** 10.1038/s41598-025-98838-8

**Published:** 2025-04-24

**Authors:** Jaakko Lampela, Niko Järveläinen, Anni Määttä, Satu Siimes, Minja Heikkilä, Hanna Wang, Jarkko Kuivalainen, Juho Liljeström, Ville Vepsäläinen, Juha Hartikainen, Seppo Ylä-Herttuala

**Affiliations:** 1 A.I. Virtanen Institute for Molecular sciences, University of Eastern Finland, Kuopio, Finland; 2 Heart Center, Kuopio University Hospital, Kuopio, Finland; 3 Gene Therapy Unit, Kuopio University Hospital, Kuopio, Finland

**Keywords:** Heart failure, Gene delivery, Experimental models of disease

## Abstract

Heart failure (HF) is a major health problem worldwide, where gene therapy could provide promising new approaches for effective treatments. Pacing-induced tachycardiomyopathy in large animals is a well-established HF model but it suffers from poor tolerability and variable outcomes. In this study, we developed a reproducible, feasible, and tolerable pacing protocol for HF gene therapy studies. Single-chamber pacemakers were implanted in pigs. HF development was monitored with trans-thoracic echocardiography (TTE), invasive left-ventricular (LV) pressure measurements, and a treadmill exercise stress test. Four weeks of rapid right-ventricular pacing at over 200 bpm produced a significant impairment of cardiac function (LV stroke volume index 11.8 ± 1.22 (mL/m^2^) at day 28 vs 18.1 ± 0.99 (mL/m^2^) at day 0, *p* < 0.01, mean ± SEM, paired t-test), and reduced exercise capacity (− 14 ± 3.19% change in 15-min treadmill exercise stress test at day 28 compared to day 0, *p* = 0.02, mean ± SEM, paired t-test). Further pacing at 150 bpm was a well-tolerated way to prevent functional recovery. Good tolerability and reproducibility of the pacing were achieved by a gradual increase in the pacing rate, no higher than 200 bpm. Global left ventricular adenoviral (Ad) gene transfer via cardiac veins was performed after three weeks of rapid pacing and found well-tolerated and feasible. In this study, the protocols of pacemaker implantation and TTE in pigs were also optimized. In conclusion, the optimized pacing protocol considerably increases the feasibility and reproducibility of the pacing-induced HF for testing of new therapeutic applications.

**Supplementary Information:**

The online version contains supplementary material available at 10.1038/s41598-025-98838-8.

## Introduction

Heart failure (HF) is a major health burden worldwide. Despite the significant advantages in HF treatments during the past decades, the prognosis of the patients with severe HF remains poor. However, new treatments need to be tested in relevant large animal models before entering clinical trials.

Pig has gained popularity as an animal model for HF because of its high anatomical and functional similarity to humans^1^. In addition, the ease of handling and suitability for long-lasting procedures under anaesthesia is a major advantage. The main disadvantage of using pigs is the somewhat limited time window when the animal’s size is suitable for imaging, handling, and procedures with commercial equipment tailored to human use.

Animal models of different types of HF, mirroring the natural variety of HF in patients, have been developed^2^. Pacing-induced, dilated cardiomyopathy-like HF has been used in pigs, sheep, and dogs^2^. Pacing-induced HF model mimics non-ischemic and non-hypertrophic HF with reduced ejection fraction (HFrEF), such as dilated cardiomyopathy, toxin-induced HF, and tachycardia-based HF. An important advantage of this model is the neurohumoral activation^3^, an important hallmark of human HF. Disadvantages include the high variation of pacing effects and partial reversibility of the HF^4^. Moreover, a notable feature of the pacing-induced HF is a functional mitral regurgitation (MR), which develops individually and significantly affects the symptoms, state, and progression of HF^5^.

Currently used pacing protocols vary a lot. In pigs, according to Paslawska et al.^6^, pacing with 170 bpm takes at least 12 weeks to reach even mild HF. In contrast, a one-week pacing of 230 bpm produces significant HF, but animals tolerate it very poorly even for a week^7^. Hàla et al.^8^ approached the problem using a gradually increasing pacing frequency, from 200 to 240 bpm. However, they still stated the problem of variable tolerance and outcome of the pacing, and recommended adjusting the pacing rate individually based on the progression of HF. This approach, however, is very subjective and obviously requires frequent measurements of the cardiac function.

In this study, we develop a reproducible, reliable, feasible, and well-tolerable HF model especially for gene therapy studies. In addition, we report here practical and comprehensive trans-thoracic echocardiography (TTE) protocol for HF studies in pigs.

## Methods

### Approval for animal experiments

All animal experiments were approved by the Animal Experiment Board in Finland and carried out in accordance with The Finnish Act on Animal Experimentation. The investigation conforms with the Guide for The Care and Use of Laboratory Animals published by the US National Institutes of Health (NIH Publication No. 85−23, revised 1996). The ARRIVE guidelines were compiled in all the used methods.

### Animal model

**Fig. 1:**
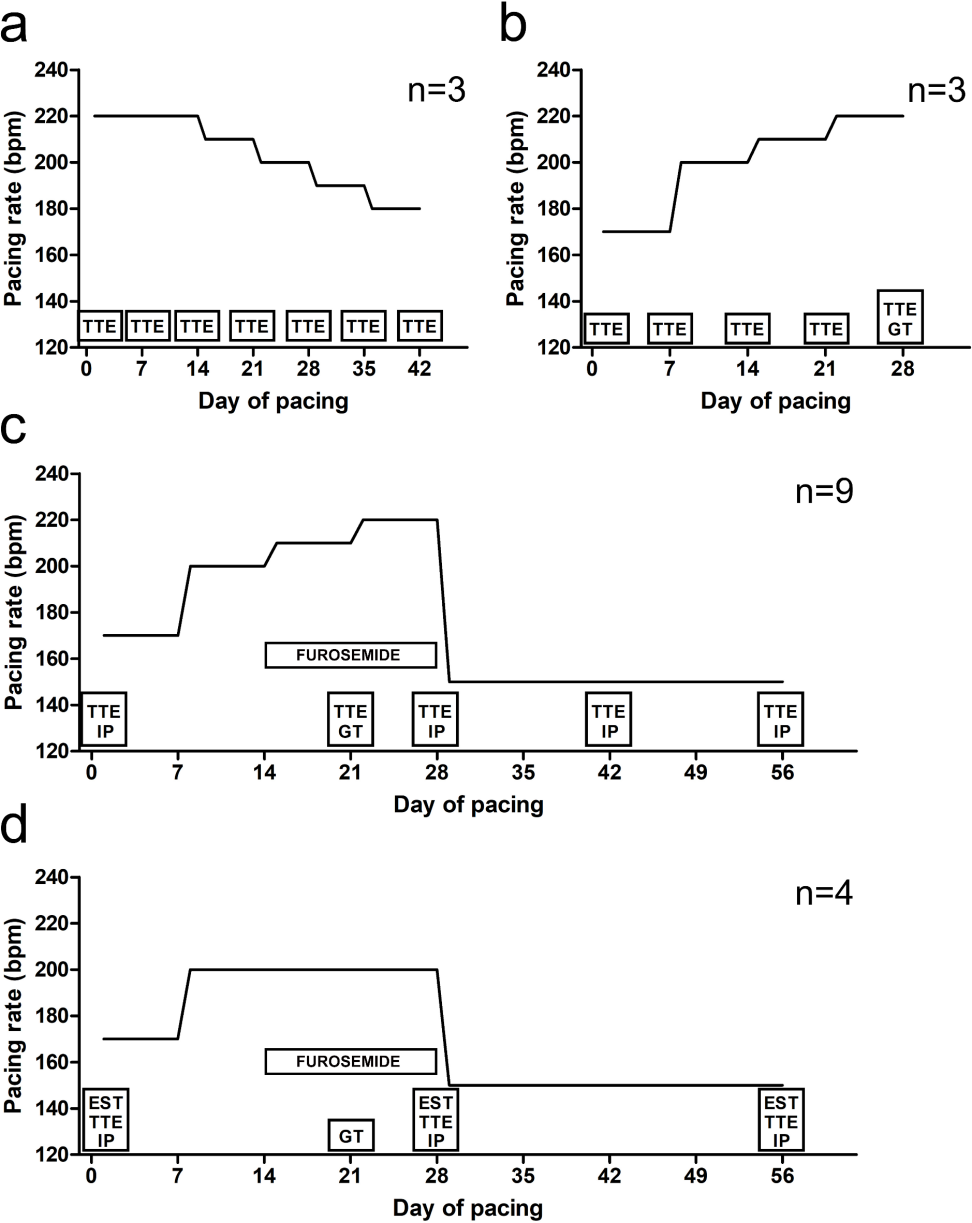
The pacing protocols of the study. (**a**) The estimated pacing curve of the protocol 1. TTE was performed immediately before start of the pacing, and weekly during the follow-up. The rate of pacing started at 220 bpm and was lowered when the pigs showed signs of decompensation of HF. (**b**) The planned pacing curve of the protocol 2. TTE was performed immediately before start of the pacing, and weekly during the follow-up. At 28th day of pacing, global LV gene transfer was simulated with saline. (**c**) The planned pacing rate curve of the protocol 3. TTE was performed immediately before start of the pacing, and further at days 21, 28, 42, and 56 of the pacing. Invasive LVEDP measurements during rest and dobutamine-induced stress were recorded immediately before pacing, and at days 28, 42, and 56 of the pacing. Global LV gene transfers with AdLacZ (2 × 10^12^ vp in total) were performed at day 21 of the pacing. (**d**) The planned pacing rate curve of the protocol (4) 15 min treadmill exercise stress test, TTE, and invasive LV pressure measurements were recorded immediately before start of the pacing, and further at days 28 and 56 of the pacing. Global LV gene transfers with AdLacZ (2 × 10^12^ vp in total) were performed at day 21 of the pacing. TTE = trans-thoracic echocardiography, GT = gene transfer procedure, IP = invasive LV pressure measurement, EST = 15 min treadmill exercise stress test.

Healthy female Finnish landrace pigs weighing 25–35 kg at the start of the experiment were used in the study. The pigs were purchased from Emolandia Oy, Finland. Sequential study design was used to develop an optimized protocol for HF (Fig. 1).

### Study overview

To establish the highest tolerated pacing rate, three pigs were paced for six weeks with the maximal generator-allowed rate, 220 bpm (protocol 1, Fig. 1a). When animals showed clinical signs of decompensated HF, such as severe fatigue, dyspnea, or lack of appetite, the pacing rate was individually decreased to the highest tolerated level. TTE was performed before pacing and weekly until the end of the follow-up. However, the need to individually adjust the pacing rate made this approach not suitable for studies with a larger number of animals.

We then subjected four pigs to receive a gradually increasing rate (protocol 2, Fig. 1b). The pacing protocol started from 170 bpm. After the first week, the pacing rate was elevated to 200 bpm, after the second week to 210 bpm, and up to 220 bpm after three weeks of pacing. If the pig did not tolerate pacing, the pacing rate was reduced to the highest tolerable level. In total, the pacing protocol lasted four weeks. TTE was performed before pacing and 7, 14, 21 and 28 days after the pacing protocol started. To assess the feasibility for gene transfer studies, we simulated a global left ventricular gene transfer procedure at day 28 of pacing. As previously reported, the gene transfer procedure was performed percutaneously via cardiac veins^9^ using saline instead of gene therapy vectors. The pacing was switched off during the procedure. In addition, a similar TTE follow-up was made on two healthy animals without a pacemaker.

Another group of twelve pigs was subjected to the gradually increasing pacing rate protocol where the pacing rate at 150 bpm was continued for another four weeks (protocol 3, Fig. 1c), aiming to maintain but not exacerbate the state of HF, as would likely happen with pacing rates higher than 170 bpm^6^. Moreover, daily 40 mg of furosemide was given p.o. during the latter two weeks of the rapid pacing phase. These pigs also underwent TTE and invasive LVEDP (left ventricular end-diastolic pressure) measurements during rest and dobutamine-induced stress before pacing and at 4, 6 and 8-week timepoints. Also, these animals underwent additional TTE and global left ventricular gene transfer at day 21 of the pacing. The gene transfer was done by retrograde coronary venous route^9^ using adenovirus (Ad) encoding beta-galactosidase (LacZ) as a transgene. A total dose of AdLacZ per animal was 2 × 10^12^ viral particles (vp).

We finally modified the stepwise protocol 3 in four pigs which were paced with a 170-bpm rate for the first week, following by three weeks of pacing at 200 bpm (protocol 4, Fig. 1d). During the latter two weeks of rapid pacing, daily dose of furosemide 40 mg was given p.o. At day 21 of pacing, global retrograde gene transfer procedures were performed, administrating 2 × 10^12^ vp of AdLacZ in total^9^. The rapid 4 weeks pacing phase was followed with another 4 weeks pacing at the rate of 150 bpm. TTE was performed at baseline, at day 28, representing the end of the rapid pacing phase, and after the moderate pacing phase at day 56 of pacing. From invasive pressure curves, clinically relevant maximal and minimal LV pressure change (+ dp/dt, − dp/dt, respectively), contractile reserve, LVESP (LV end-systolic pressure) and LVEDP were measured in rest and dobutamine-induced stress. In addition, these pigs were subjected to a 15 min treadmill exercise stress test^10^ in order to study the feasibility of measuring functional changes in the performance of the HF pigs.

Sample sizes for each protocol in this iterative study were decided one by one, based on tolerability results obtained from the previous protocol. Following exclusion criteria were established a priori: Signs of pacemaker-related bacterial infection, significant heart defect at the baseline echocardiography, and significant health problem unrelated to the study protocol. Moreover, if signs of leg-related health problems were observed, the animal in question was not subjected to the treadmill exercise stress test.

### Anesthesia and medication

Animals were sedated with intramuscular administration of xylazine 0.2 ml/kg and midazolam 0.2 ml/kg. General anaesthesia was achieved with propofol with individually adjusted infusion rate and fentanyl 10 ug/kg/h i.v. Animals were intubated and mechanically ventilated.

Prophylactic doses of 1000 mg cefuroxime twice a day were given intramuscularly from the morning of the implantation until the fifth postoperative day. To reduce postoperative pain, the pacemaker pocket and lead tunnel regions were locally anaesthetized during the implantation. Moreover, animals received buprenorphine 0.3 mg i.m. three times a day and carprofen 4 mg/kg i.m. once a day until the third postoperative day, or longer if the animal showed any signs of pain.

Before the gene transfer and intraventricular pressure measurement procedures, pigs received prophylactic cefuroxime 500 mg i.m. To prevent arrhythmias during gene transfer, animals were given 100 mg of lidocaine i.v. and 615 mg of MgSO4 i.v. 30 mg of enoxaparin i.v and isosorbide dinitrate s.l. were administered after the insertion of the introducer sheaths. For postoperative analgesia, buprenorphine 0.3 mg i.m. or/and carprofen 4 mg/kg i.m. were given. At the end of the experiment, animals were sacrificed with saturated potassium chloride solution i.v, after a terminal load of propofol.

### Pacemaker implantation

A single-chamber pacemaker (Assurity MRI 1727, Abbott, USA) was used for pacing. The pacemaker implantation procedure was performed under general anaesthesia and sufficient monitoring. Strict aseptic procedures were followed during the operations.

#### Lead positioning and testing

The lead-introducing phase was performed while the animal was lying on its back. Under fluoroscopic guidance, 100 cm pacing lead was carried to the cardiac right ventricle (RV) via a standard 6 F splittable introducer sheath in the pig’s external jugular vein. With the support of soft or extra soft curved in-lead guidewire, the lead tip was screwed to the anterior interventricular septum (IVS) near the RV outflow tract, which is the most sensitive area for pacing in the pig’s RV, according to the authors’ experience.

The lead was first screwed with five rounds to the target endocardial site. Then, the positioning was tested using dispensable pacing system analyzer cables (Abbott) connected to the programmer device (Abbott). Stable attachment of the lead was ensured by additional ten screw rounds when the following criteria were met: (1) sensed voltage over 3 mV, (2) lead impedance between 300 and 1500 Ω, (3) pacing threshold 1 V or lower, (4) visible current of injury in the endocavitary electrogram.

After assured lead attachment, the puncture-related skin incision was lengthened to 2 cm and using blunt dissection, the underlying muscle membrane was revealed. Then, the in-lead guidewire and the introducer sheath were removed, and a plastic collar was strangled around the lead stem with tight knots using unabsorbable suture material. The collar was attached to the muscle membrane beside the vein puncture site with semi-loose, unabsorbable stitches positioned to ensure sufficient hemostasis of the puncture.

Approximately 40 cm of the lead was left outside, and the rest was pushed in, under fluoroscopic control. The extra length of the lead in the animal was guided to make loops in the cardiac atrium and proximal vena cava, allowing the animal to grow without the risk of the lead detachment. According to the author’s experience, a long loop deep in the inferior vena cava can spontaneously attach to the vascular wall during the pig’s growth.

#### Pacemaker positioning and lead tunneling

After the lead implantation phase, the pig was turned on its side, opposite to the punctured vein. About 7 cm incision was made in the direction of the skin folds, posterior to a line from the ear to the superior angle of the scapula. Via the incision, a tissue pocket slightly larger than the pacemaker device was made using blunt dissection of the tissue layers. With blunt instruments, the lead was subcutaneously tunneled from the puncture site to the pacemaker pocket and attached to the pacemaker device. Then, the pacemaker was interrogated, and sense voltage, lead impedance, and pacing threshold were measured, assuring the lead positioning and the pacemaker device function. Then, the pacemaker device was attached to muscle membrane at the bottom of the pocket with a loose, unabsorbable stitch. The extra length of the lead was looped under the pacemaker, allowing the animal to grow without the risk of detachment or stretch of the lead.

Initially, the pacemaker pocket was made subcutaneously. The closing was done with three layers: The pacemaker pocket and the subcutaneous tissue layer were closed by layers of absorbable sutures, and the skin was closed with non-absorbable sutures. Later, we added a gentamicin-collagen implant to surround the pacemaker, and non-absorbable suture layers were sutured knots downwards. The pacemaker pocket was also made below the most superficial muscle layer, allowing one more layer of absorbable sutures and leaving the skin without any pressure regardless of the animal behavior.

Postoperatively, the wounds were cleaned with iodized povidone 100 mg/ml, and the wound dressings were changed every other day. The pens were kept as clean as possible during the wound healing. The skin sutures were removed on the tenth postoperative day.

#### Programming of the device

Programming to the pacemakers was done using Merlin™ Patient Care System (Abbott). After pacemaker implantation, pacemakers were programmed to ‘VVI’ mode, allowing a 30 bpm rate. After at least one week of post-operative healing, pacemakers were switched to ‘VOO’ mode at a rate of 170 bpm. Higher pacing rates required access to the ‘special functions’ programming, which was allowed and supported by the manufacturer’s (Abbott) technical support. Pacing-mediated heart rate was checked with ECG, and the appropriateness of the pacing parameters was checked in every interrogation. Pacing voltage was set at 2.5 V. Before any measurements or procedures, pacemakers were programmed to ‘Pacing off’ mode. High-rate pacing was switched back on only after the animal had spontaneously stood up after anaesthesia.

### Trans-thoracic echocardiography (TTE)

**Fig. 2:**
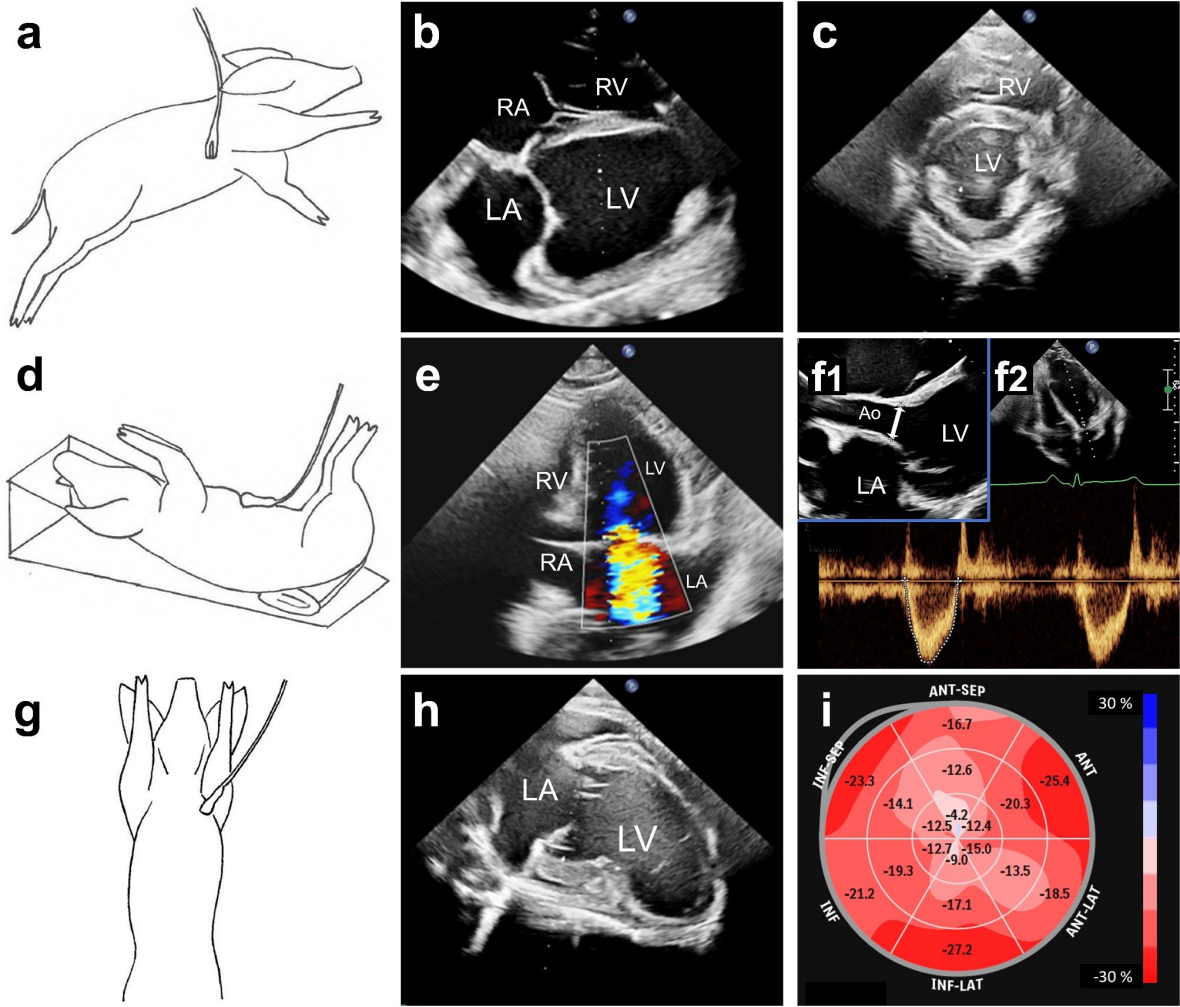
TTE was performed via three trans-thoracic windows. (**a**) Transducer positioning for parasternal four-chamber, three-chamber and short-axis views. (**b**) Parasternal four-chamber view, (**c**) Parasternal short-axis view, (**d**) Transducer and pig positioning for apical views. (**e**) MR was assessed via apical four-chamber view. (**f1**) LVOT was measured via prasternal long-axis view. The white arrow illustrates the measuring line. (**f2**) LVOT systolic velocity-time integral was measured via subcostal window. (**g**) Transducer positioning for parasternal two-chamber view. (**h**) parasternal two-chamber view. (**i**) Bulls eye map of LV global strain. LV = the left ventricle, RV = the right ventricle, LA = the left atrium, RA = the right atrium, Ao = aorta.

The state of cardiac function was assessed by TTE under general anesthesia (Philips EPIQ Elite Ultrasound system for General imaging, Release 5.0.1, Philips Heath Systems, Bothell Washington, USA). Thorough assessment was achieved via three transthoracic windows: Four-chamber and three-chamber long-axis views were obtained from 4th or 5th right intercostal space when the animal was lying left side down (Fig. 2a, b, f1). By rotating the transducer 90°, the same intercostal windows allowed obtaining short-axis sequence records from the basal, papillary, and apical levels of LV (Fig. 2a, c). The two-chamber view was obtained from 2nd or 3rd left parasternal intercostal space when the animal was lying on its back (Fig. 2g, h). Apical four-chamber or three-chamber views were achieved from para-xiphoidal subcostal windows, when the animal was lying on its back. The ventricular foreshortening in the apical views, which is typical for pigs, could be alleviated by placing the pig on an inclined plane with the head side up and at the same time bending its caudal end ventrally (Fig. 2d).

End-diastolic diameters of the LV, RV and IVS were measured with M-mode of parasternal long axis view at the opened mitral valve leaflet tips level. Dynamic acquisitions of modified two-chamber (Fig. 2h) and four-chamber (Fig. 2b) views are taken from parasternal windows for bi-plane LV ejection fraction (LVEF) and volume assessments. Left atrial (LA) volume was estimated by measuring the LA area from the parasternal four-chamber view. Global LV longitudinal strain analyses were made using parasternal two-chamber, three-chamber and four-chamber sequences. Global LV circumferential strain was calculated from dynamic acquisitions of short axis views at the levels of mitral valve, papillary muscles, and the RV apex. The strain and bi-plane LVEF analyses were calculated using Philips Q-lab (aCMQ) software version 13.0 (Philips Health Systems, Bothell Washington, USA).

The area of the LV outflow tract (LVOT) was measured from a modified parasternal long-axis view (Fig. 2f1). From the apical three-chamber view, the velocity-time integral (VTI, cm/s) of systolic LVOT blood flow was recorded with pulsed wave (PW) doppler mode (Fig. 2f2). LV stroke volume was calculated as the product of LVOT area and LVOT VTI. Due to the pig growth during the follow-up, LV stroke volume and chamber volumes were indexed by dividing them by estimated body surface area, calculated as body weight multiplied by 0.0734 m^2^/kg^1^ (LV stroke volume index, LV SVI, LA volume index, LAVI, LV end-diastolic volume index, LV EDVI).

Diastolic blood flow through the mitral valve was acquired with PW doppler from the apical four-chamber view, and the maximal velocities of the E and A waves were measured. With PW tissue-doppler imaging mode, diastolic motion waves of the medial and lateral mitral annulus were acquired, and an average of the lateral and medial e’ waves was calculated. Moreover, times of isovolumic systole, ejection and isovolumic relaxation (IVCT, ET and IVRT, respectively) were measured from the mitral annular tissue doppler curve, and the Tei index was calculated.

Mitral valve function was evaluated from magnified apical four-chamber view. If present, the severity of MR was assessed with measurements of vena contracta (MR VC) and effective regurgitant orifice area (MR EROA). MR VC was measured from colour doppler acquisition from the area of the mitral valve. PISA method^11^ was used for EROA calculation.

Aortic valvular diseases were excluded with visual assessment of the aortic valve function via intercostal and subcostal windows.

### Invasive LV pressure measurements

Under fluoroscopic guidance and following strict aseptic procedures, a pigtail catheter was advanced to the LV via an introducer sheath in the femoral artery. The pressure sensor was calibrated to the air pressure before the measurement and was kept at the heart height during the measurement. The pressure graph was printed out and graphically analyzed. After the pressure measurements at rest, an infusion of dobutamine 20 µg/kg/min was started. At 5 min from the infusion’s start, the pressure graph of LV was printed and analyzed similarly.

### Gene transfer procedure

In order to investigate the feasibility of global LV gene transfer via cardiac veins in pigs with significant HF, saline or 2 × 10^12^ vp of AdLacZ as biologically inactive transgene was administered as described previously^9^. Briefly, the anterior cardiac vein, the middle cardiac vein and the most prominent vein of the LV lateral wall were cannulated with 4-F wedge pressure balloon catheters, and retroinjections of saline of AdLacZ were administered in the veins one by one. While the retroinjection was administered, both the corresponding artery and the most crucial anastomotic vein of the retroinjected vein were transiently occluded with balloon catheters for a maximum of 60 s.

### Sampling

After sacrifice, tissue samples from the heart, lung, liver, spleen, kidney, ovary and para-aortal lymph node were collected. Tissue samples were immersion fixed for 48 h in 4% paraformaldehyde and further in 15% sucrose, before embedding in paraffin^12^.

### Statistics

To compare LVEF results of paced pigs to healthy ones, non-paired t-test was used. To compare cardiac function results to baseline measurements, paired t-test was used. To compare the tolerability of the HF induction protocols with each other, the Log-rank (Mantel-Cox) test was used. Computations were performed with Prism 5 version 5.03 (GraphPad Software, California, USA).

## Results

### Pacemaker-related infections

Despite strict following of aseptic procedure, antibiotic prophylaxis and adequate postoperative care, bacterial infections of the pacemaker pocket and the lead tunnel caused exclusion of one pig subjected to protocol 2 and three pigs subjected to protocol 3. Observed potential causes of infections included suture material pushing out through the wound, and skin ulcers at the pacemaker site, which were probably related to the pig’s behaviour of rubbing the wound area. Subcutaneously inserted pacemakers were susceptible to infections, even with a gentamicin-collagen implant added surrounding the device. Finally, implanting the pacemaker device below the most superficial muscle layer and making subcutaneous sutures knots downwards led to avoidance of infections within the protocol 4.

### Cardiac function and the pacing protocol tolerance

**Fig. 3:**
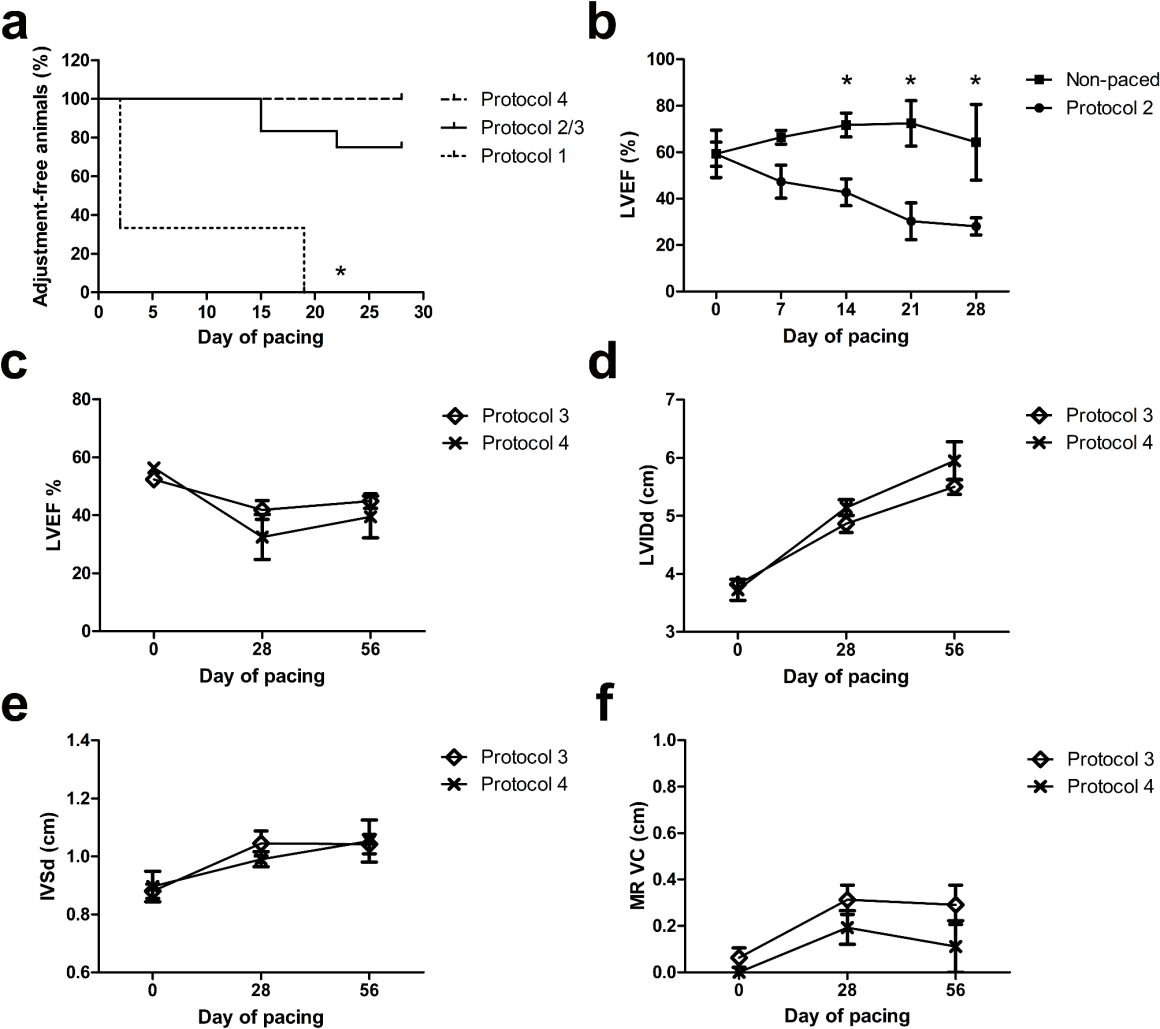
(**a**) The percentage of animals free from pacing rate adjustment in HF induction. Protocols 2 and 3 were combined because of their similarity in rapid pacing phase. The tolerability of protocol 1 differed significantly from protocols 2/3 and 4 (*p* = 0.001 and *p* = 0.01, respectively, Log-rank test). Difference in adjustment-free survival between protocols 2/3 and 4 was not statistically significant. (**b**) LVEF measurements of pigs paced according to the protocol 2 (*n* = 3), compared to pigs without pacing (*n* = 2). * = *p* < 0.05, unpaired t-test. (**c**) Measured LVEF (%) in pigs paced according to protocols 3 and 4. There were no statistically significant differences between the groups in any presented timepoint (unpaired t-test). (**d**) Measured LVIDd (cm) in pigs paced according to protocols 3 and 4. There were no statistically significant differences between the groups in any presented timepoint (unpaired t-test). (**e**) Measured IVSd (cm) in pigs paced according to protocols 3 and 4. There were no statistically significant differences between the groups in any presented timepoint (unpaired t-test). (**f**) Measured VC of mitral regurgitation in pigs paced according to protocols 3 and 4. There were no statistically significant differences between the groups in any presented timepoint (unpaired t-test).

In the protocol 1, the tolerance for pacing with 220 bpm varied a lot, leading to many individual adjustments for the pacing rate (Fig. 3a). Only one of the animals tolerated pacing of 220 bpm until the 18th day of pacing; then, the rate was decreased due to fatigue and dyspnea. Other animals did not tolerate the 220-bpm rate at all. The frequent need to individually adjust the pacing rate and thus variable outcomes made this approach not suitable for studies with a larger number of animals.

Of the three non-infected pigs subjected to the protocol 2, the gradually increasing pacing rate was tolerated significantly better compared to the protocol 1 (Fig. 3a). Two of the three animals tolerated the protocol 2 as planned, whereas one pig needed pacing rate reduction to 200 bpm in the fourth week. The LVEF of the paced animals lowered congruently, whereas the LVEF of non-paced animals remained stable (Fig. 3b). However, the deep anesthesia necessary for the gene transfer procedure could not be induced without acute escalation of HF in two of the three paced animals at day 28 of pacing.

Of the nine non-infected pigs subjected to the protocol 3, two animals needed pacing rate reduction during the rapid pacing phase due to signs of HF decompensation. However, the pacing rate of both animals could be maintained at or above 200 bpm until the end of the rapid pacing phase. Moreover, one animal was not paceable in rates over 200 bpm due to a limiting individual refractory period: even though impedance and pacing threshold remained at adequate levels, a higher pacing rate did not lead to adequately higher heart rate, not even with a higher pacing voltage. All nine animals tolerated the gene transfer procedure. The four-week moderate pacing phase from day 29 to 56 was well tolerated and allowed animals to recover while keeping the cardiac function significantly below the baseline level. (Fig. 3a, c–f; Table 1).

**Table 1:** The cardiac function measurement results of the animals subjected to the protocol 3 (n = 9).

Timepoint	D0	D21	D28	D42	D56
	mean ± SEM	mean ± SEM	mean ± SEM	mean ± SEM	mean ± SEM
Body weight (kg)	27.1 ± 0.89	47.2 ± 1.29**	51.2 ± 1.40**	62.7 ± 1.41**	74.0 ± 2.33**
Heart rate at rest (bpm)	102 ± 3.38	86.7 ± 3.99*	99.1 ± 6.12 ns	89.6 ± 3.37*	80.8 ± 4.65**
IVSd (cm)	0.88 ± 0.02	0.92 ± 0.04*	1.04 ± 0.04**	1.06 ± 0.04**	1.04 ± 0.03**
LVIDd (cm)	3.82 ± 0.06	5.21 ± 0.09**	4.86 ± 0.15**	5.30 ± 0.14**	5.50 ± 0.13**
RVIDd (cm)	2.48 ± 0.04	3.15 ± 0.17**	3.15 ± 0.13**	3.26± 0.12 **	3.68 ± 0.13**
LVEF (%)	52.3 ± 1.75	44.1 ± 2.65*	41.8 ± 3.22**	43.7 ± 1.45**	44.9 ± 2.49**
Global longitudinal strain (%)	− 24.2 ± 1.47	− 19.3 ± 1.52*	− 18.8 ± 1.52*	− 18.5 ± 1.00 **	− 20.3 ± 1.51 ns
Global circumferential strain (%)	− 19.2 ± 1.07	− 16.3 ± 1.11 ns	− 15.3 ± 1.39*	− 16.1 ± 0.55 *	− 18.3 ± 1.26 ns
LV SVI (mL/m^2^)	21.1 ± 1.95	17.7 ± 1.49 ns	16.6 ± 1.30 ns	16.0 ± 0.75*	16.0 ± 0.75 ns
LV EDVI (mL/m^2^)	29.2 ± 1.04	34.4 ± 1.68*	31.7 ± 1.35 ns	33.7 ± 1.89 ns	28.4 ± 1.30 ns
MR EROA (cm^2^)	0.00 ± 0.00	0.03 ± 0.01*	0.04 ± 0.01*	0.03 ± 0.01 ns	0.03 ± 0.02 ns
MR VC (cm)	0.06 ± 0.04	0.29 ± 0.05**	0.31 ± 0.06**	0.25 ± 0.09 ns	0.29 ± 0.08*
LAVI (mL/m^2^)	12.5 ± 1.13	21.7 ± 2.05**	16.1 ± 1.67 ns	15.2 ± 2.11 ns	14.5 ± 1.44 ns
Mean e’ (cm/s)	7.92 ± 0.36	9.72 ± 0.83 ns	7.47 ± 0.42 ns	8.31 ± 0.62 ns	8.77 ± 0.45 ns
E/A ratio	1.07 ± 0.05	1.40 ± 0.09*	1.26 ± 0.11 ns	1.38 ± 0.10**	1.23 ± 0.10 ns
E/e' ratio	8.21 ± 0.26	8.58 ± 0.68 ns	9.40 ± 0.55 ns	9.39 ± 0.78 ns	8.06 ± 0.48 ns
IVCT (ms)	59.5 ± 4.28	65.8 ± 6.64 ns	74.4 ± 8.01 ns	85.6 ± 5.18**	87.0 ± 8.07*
ET (ms)	238 ± 8.23	241 ± 11.9 ns	216 ± 9.70 ns	235 ± 6.4 ns	249 ± 8.06 ns
IVRT (ms)	89.0 ± 2.29	85.6 ± 11.0 ns	109 ± 5.78**	90.1 ± 7.91 ns	93.2 ± 4.02 ns
Tei index	0.63 ± 0.04	0.66 ± 0.11 ns	0.88 ± 0.09 ns	0.75 ± 0.05 ns	0.74 ± 0.06 ns
LV EDP at rest (mmHg)	15.0 ± 1.66		16.5 ± 3.16 ns	23.3 ± 1.48*	22.3 ± 2.13*
LV EDP at stress (mmHg)	11.7 ± 2.42		9.86 ± 4.02 ns	19.3 ± 3.54 ns	24.0 ± 3.16*

The measurement results of D21, D28, D42, and D56 were compared to measurement results of D0, and paired t-test was used to show statistical significance of the change. ** = *p*-value < 0.01, * = *p*-value < 0.05, ns = non significant, indicating *p*-value at or over 0.05.

With the protocol 3 pacing, the cardiac systolic function, evaluated with LVEF, SVI, and global strain, was impaired congruently with the results of the protocol 2. Overpacing caused cardiac enlargement, leading to regurgitation in atrioventricular valves (Figs. 2e and 3d and f; Table 1), varying from mild to severe. Aortic valve dysfunctions were not detected. LVEDP elevated significantly during the follow-up, indicating diastolic dysfunction. However, no myocardial fibrosis, often linked to elevated LVEDP, was detected in histological assessment. E/e’ ratio did not correlate with LVEDP, as it does in humans^13^. (Fig. 3c–f; Table 1)

With the protocol 4, all pigs tolerated the pacing well and no adjustments in the pacing rate were needed (Fig. 3a). The cardiac function developed congruently with protocols 2 and 3, even though the pacing rate was lower during the last two weeks of the rapid pacing phase (Fig. 3c–f; Table 2). As a clinically relevant endpoint measurement, the 15-min treadmill exercise stress test was well tolerated. The exercise stress test results lowered significantly due to the pacing. The absolute values −dp/dt and + dp/dt at rest were lowered after the rapid pacing phase. However, during dobutamine-induced stress, the absolute values of both measures grew regardless of the pacing, forming a growing contractile reserve. (Table 2)

**Table 2:** Cardiac function and treadmill execrise stress test results of pigs underwent the protocol 4 of pacing (*n* = 4).

Timepoint	D0	D28	D56
	mean ± SEM	mean ± SEM	mean ± SEM
Body weight (kg)	30.8 ± 2.24	51.1 ± 2.14**	76.6 ± 1.73**
Heart rate at rest (bpm)	107 ± 4.17	95.3 ± 4.77 ns	94.0 ± 6.67 ns
RVIDd (cm)	2.16 ± 0.17	3.58 ± 0.37*	3.71 ± 0.37*
LV Global longitudinal strain (%)	− 20.7 ± 0.96	− 12.1 ± 3.09 ns	− 16.7 ± 3.11 ns
LV Global circumferential strain (%)	− 26.7 ± 1.25	− 16.9 ± 3.19 ns	− 17.7 ± 3.60 ns
LV SVI (mL/m^2^)	18.1 ± 0.99	11.8 ± 1.22**	14.0 ± 1.00 ns
LV EDVI (mL/m^2^)	25.4 ± 2.06	33.9 ± 5.19 ns	38.6 ± 6.60 ns
MR EROA (cm^2^)	0.000 ± 0.000	0.005 ± 0.003 ns	0.013 ± 0.013 ns
MR VC (cm)	0.00 ± 0.00	0.19 ± 0.07 ns	0.11 ± 0.11 ns
LAVI (mL/m^2^)	9.86 ± 1.56	14.9 ± 5.32 ns	14.6 ± 2.48 ns
Mean e’ (cm/s)	8.05 ± 1.07	8.63 ± 1.61 ns	8.43 ± 0.88 ns
E/A ratio	1.05 ± 0.05	1.33 ± 0.19 ns	1.50 ± 0.19 ns
IVCT (ms)	60.0 ± 9.05	85.3 ± 3.47 ns	99.8 ± 11.0 ns
ET (ms)	214 ± 7.2	212 ± 18.3 ns	218 ± 9.3 ns
IVRT (ms)	101 ± 8.0	68.5 ± 5.6 ns	78.0 ± 5.9 ns
Tei index	0.76 ± 0.10	0.74 ± 0.06 ns	0.82 ± 0.06 ns
LV EDP at rest (mmHg)	15.8 ± 1.44	34.5 ± 5.12*	33.8 ± 2.25**
LV EDP at stress (mmHg)	18.0 ± 3.24	38.3 ± 5.11 ns	32.3 ± 2.25*
LV ESP at rest (mmHg)	101 ± 5.12	101 ± 7.19 ns	110 ± 7.89 ns
LV ESP at stress (mmHg)	99.0 ± 7.45	133 ± 3.95*	150 ± 2.87*
+ dp/dt max at rest (mmHg/s)	1628 ± 82.5	1238 ± 97.5 ns	1440 ± 156 ns
+ dp/dt max at stress (mmHg/s)	2255 ± 320	2573 ± 320 ns	3439 ± 76.1 ns
Contractile reserve (%)	39.0 ± 26.4	105.3 ± 17.7 ns	134 ± 21.1 ns
−dp/dt min at rest (mmHg/s)	− 1680 ± 106	− 1313 ± 183 ns	− 1793 ± 191 ns
−dp/dt min at stress (mmHg/s)	− 1905 ± 133	− 2203 ± 341 ns	− 2875 ± 350 ns
15 min exercise stress test change (%)	0 ± 0	− 14.6 ± 3.19*	− 17.1 ± 4.23*

The measurement results of D28 and D56 were compared to measurement results of D0, and paired t-test was used to show statistical significance of the change. ** = *p*-value < 0.01, * = *p*-value < 0.05, ns = non significant, indicating *p*-value at or over 0.05.

## Discussion

In this study, we aimed to establish and optimize a large animal HFrEF model, especially regarding tolerability and repeatability, and to reduce unnecessary exclusions of animals in future studies. Since the basic principles and pathophysiology of this model are well-established and reported previously^14^, the focus of this study was to investigate and optimize the practical protocol for HF induction and follow-up, especially from the aspect of gene therapy studies. According to our experience, the rapid growth of pigs challenges the implementation of studies lasting longer than three months, especially when advanced imaging or an exercise stress test is needed. Thus, we designed the HF induction protocol to be as compact as possible while still respecting the goals of keeping the protocol reproducible and tolerable and having significant existing HF before the gene therapy is administered. Useful contributions of this report also include detailed descriptions of TTE and RV leaded pacemaker implantation in pigs. As a limitation of this study, the pathophysiologic processes underlying this HF model, including neurohumoral, electrical or cellular remodelling, were not investigated.

Stepwise raising of the pacing rate is the way to achieve tolerability and reproducibility of the efficient pacing protocol. Initial stepwise pacing protocol led a week-to-week reduction of the cardiac function. There is clearly tolerability challenges related to pacing rates over 200 bpm in pigs, which observation is also supported by reports of previous studies using higher rates^7,8^ Moreover, we observed that a pacing rate over 205–215 does not lead to a corresponding increase in the heart rate in some pigs. In our lab, this phenomenon has been observed in about 10% of landrace pigs (data not shown). Therefore, in order to minimize wasteful animal exclusions and the need of individual adjustments in the pacing protocol, we recommend pacing no higher than 200 bpm, which was still shown to be an effective rate for the induction of HF. Furosemide medication is justified in order to prevent tolerability challenges related to abnormal fluid load when HF is at its most severe. Fluid intake restriction, which could increase the animal’s suffering, was not considered necessary and was refrained from.

TTE is a feasible and comprehensive way to measure cardiac function in pigs. The main pitfall lies in the variable heart rate in pigs which, however, can mainly be overcome by keeping the measurement situation calm, ensuring that the animals are sufficiently hydrated and under sufficiently deep anaesthesia. Importantly, even a severe HF did not prevent the treadmill exercise stress test from being safely performed.

This study employed adenovirus gene transfer because of its known efficiency and safety in cardiac delivery^9,15^. The gene transfer was timed so that the highest transgene expression would be matched with the timepoint of HF being at its worst. When using vectors with different kinetics, the timing of the gene transfer should be optimized for each vector type. The day 28 of pacing proved to be a too late timepoint for procedures under deep anesthesia.

The pacing-promoted impairment in the cardiac function varies a lot between individual pigs, being impossible to estimate beforehand. Thus, we recommend doing baseline measurements in a gene therapy study as late as possible, but still respecting the transgene expression kinetics of the employed vector. Especially the functional MR develops individually over time and should be caught on baseline measurements since it significantly affects the HF state^5^.

Based on the clear experience over the years in our lab, we have decided to prefer female pigs. Male pigs are more aggressive, and the presence of both sexes would make coexistence in experimental animal facilities more difficult. Using only one sex also reduces biological variation. Noteworthy, this is also likely to play a role in the results of this study, as the literature suggests that males are more susceptible to developing HFrEF^16,17^.

The main limitations of this model are the absence of myocardial fibrosis and the reversibility of cardiac function, which was underlined by the shown elevated contractile reserve of the failing hearts. Moreover, pigs are usable only at rapidly growing age, which limits the long-term follow-up and raises the problem of a significant regeneration capacity. However, by adding well-tolerable moderate pacing at 150 bpm to the follow-up, the reversibility problem can be mostly alleviated.

Pig’s natural behaviour leads to increased exposure to bacteria, which in turn leads to an increased possibility of wound infections, especially when implanted material is needed. However, pacemaker-related infections can mainly be avoided by minimizing all sources of bacterial exposure to the wound. Crucial manoeuvres to avoid the infections were implanting the pacemaker below the most superficial muscle layer and suturing knots downwards in the subcutaneous tissue layers.

In conclusion, the pacing-induced pig HF model is a feasible platform for gene therapy studies when the following is met: (i) pacing is elevated gradually but not higher than 200 bpm, (ii) pacemaker-related infections are avoided, (iii) baseline measurements of the study are made no earlier than two weeks from the start of the pacing, (iv) invasive gene transfer procedure is made at a tolerable timepoint. We recommend no later than three weeks from start of the pacing, and (v) moderate pacing at 150 bpm is kept up during the follow-up.

## Electronic supplementary material

Below is the link to the electronic supplementary material.

Supplementary Material 1

## Data Availability

All data generated or analyzed during this study are included in this article and supplementary material.
